# Vaccination and anaphylaxis: a forensic perspective

**DOI:** 10.3325/cmj.2017.58.14

**Published:** 2017-02

**Authors:** Cristian Palmiere, Camilla Tettamanti, Maria Pia Scarpelli

**Affiliations:** 1 CURML, University Center of Legal Medicine, Lausanne 25, Switzerland; 2 Department of Legal Medicine, University of Genova, Genova, Italy

## Abstract

**Aim:**

To review the available literature pertaining to fatalities following vaccine administration and, in particular, cases of vaccine-related fatal anaphylaxis.

**Method:**

The MEDLINE database was systematically searched up to March 2016 to identify all relevant articles pertaining to fatal cases of anaphylaxis following vaccine administration.

**Results:**

Six papers pertaining to fatal anaphylaxis following vaccination were found relevant. Mast cell tryptase and total IgE concentration was assessed exclusively in one case. Laryngeal edema was not detected in any of these cases, whereas eosinophil or mast cell infiltration was observed in lymphoid organs. In one case, immunohistochemical investigations using anti-tryptase antibodies allowed pulmonary mast cells and degranulating mast cells with tryptase-positive material outside to be identified.

**Conclusion:**

In any suspected IgE-mediated fatal anaphylactic cases, biochemical investigations should be systematically performed for forensic purposes. Splenic tissue should be routinely sampled for immunohistochemical investigations in all suspected anaphylaxis-related deaths and mast cell/eosinophil infiltrations should be systematically sought out in the spleen, myocardium, and coronary artery wall. The hypothesis of fatal anaphylaxis following vaccination should be formulated exclusively when circumstantial data, available medical records, laboratory investigations, and autopsy or histology findings converge in a consistent pattern. The reasonable exclusion of alternative causes of death after all postmortem investigations is also imperative in order to establish or rule out a cause-and-effect relationship between vaccine administration and any presumptive temporarily-related death.

Vaccination is considered one of the greatest achievements of public health, with vaccination programs contributing to the decline in mortality and morbidity of various infectious diseases. The high rate of childhood vaccination in most developed countries indicates that it remains a widely accepted public measure (1).

Although coverage levels for most childhood vaccines remain high, numerous studies have documented that vaccine-related confidence has been decreasing among parents over the past several years. Vaccine hesitancy refers to concerns regarding vaccine safety and necessity. Nonetheless, most vaccine-hesitant parents do proceed with vaccinations, albeit often with a delay in some or all vaccines that potentially leave their children at risk for vaccine-preventable diseases. Parents who refuse all recommended vaccines are relatively rare (1-5).

Access to vaccine information and misinformation from a wide range of sources significantly influences vaccine decision-making. Parents may hear a multitude of messages regarding vaccination, some of which conflicting or inaccurate. This may lead to misperceptions that can influence vaccine acceptance. Negative media coverage about vaccine safety or vaccine-related illnesses, injuries or death may correlate with an increased incidence of vaccine-preventable diseases (1,5).

Hundreds of millions of vaccinations are administered to children and adults in the world. Serious adverse reactions, including anaphylaxis, are uncommon. However, temporarily associated deaths can occur following immunization and these require careful postmortem assessment. It is essential to establish whether the death is coincidental or causally related to the administered vaccination in order to avoid social concerns that may negatively impact public perception or vaccine acceptance, if not the actual decision to adhere to vaccination programs or not (6).

Even though virtually all vaccines have the potential to trigger anaphylaxis, the risk of anaphylaxis after vaccination is extremely low and varies with different vaccine types (7,8).

Forensic pathologists may occasionally encounter cases of deaths following vaccinations or possibly causally related to vaccination due to the unexplained nature of death and the potential relationship to the adverse effects of the administered vaccine.

There have been very few cases of vaccine-related fatal anaphylaxis described in forensic literature. Of those reported, only a small part had undergone the exhaustive postmortem investigations necessary to correctly formulate the hypothesis of anaphylaxis-related death following vaccine administration. These include total and specific IgE assessment, mast cell tryptase determination and histological/immunohistochemical examination (9).

The aim of this study was to review the available literature pertaining to fatalities following vaccine administration and, in particular, cases of vaccine-related fatal anaphylaxis. These deaths were considered with respect to occurrence, patient characteristics, administered vaccine and performed biochemical or histological investigations as well as any other useful information identified based on clinical history and medical record review.

## MATERIAL AND METHODS

The MEDLINE database was systematically searched up to March 2016 to identify all relevant articles pertaining to fatal cases of anaphylaxis following vaccine administration. The search strategy used the keywords and/or mesh-terms “anaphylaxis”, “anaphylactic”, “anaphylactoid”, “shock”, “allergy”, “allergic”, “nonallergic”, “non-allergic”, “hypersensitivity”, “immediate”, “reaction(s)”, “adverse reaction(s)”, “side effect(s)” “adverse effect(s)”, “fatal”, “fatality”, “fatalities”, “lethal”, “death”, “forensic”, “medicolegal”, “medico-legal”, “tryptase”, “beta-tryptase”, “β-tryptase”, “mast cell tryptase”, “postmortem”, “post-mortem” or “autopsy” combined with any combination of the following: “vaccine(s)” (“influenza”, “pertussis”, “measles”, “mumps”, “rubella”, “pneumococcus/pneumococcal”, “meningococcus/meningococcal”, “haemophilus”, “encephalitis”, “tuberculosis”, “hepatitis”, “poliovirus”, “poliomyelitis”, “polio”, “rabies”, “typhoid”, “cholera”, “varicella”, “yellow fever”, “zoster”, “tetanus”, “diphtheria”, “toxoid(s)”, “hexavalent”, “trivalent”, “quadrivalent”, “pentavalent”, “monovalent”, “papillomavirus”, “rotavirus”), “vaccine components”, “vaccination(s)” and “immunization(s)”. The reference lists of the selected articles were then hand-searched for additional, potentially relevant articles. In order to be considered significant for the aim of our study, articles had to report fatal anaphylactic reactions following vaccine administration, patient characteristics (age, gender, medical record availability) and vaccine details. The absence of autopsy/histology findings or postmortem biochemical investigation results was not considered exclusion criteria. Published review articles pertaining to fatal reactions to vaccinations were included when data concerning individual fatal cases could be obtained. Two reviewers [CP and CT] checked all potentially relevant data in order to select pertinent papers. Of all the latter, full text papers were retrieved for further checking of inclusion and exclusion criteria. Articles that did not mention patient characteristics (age, gender, medical records) and vaccine type were excluded. Inclusion and exclusion criteria were checked independently by two reviewers (CT and MPS).

**Figure 1:**
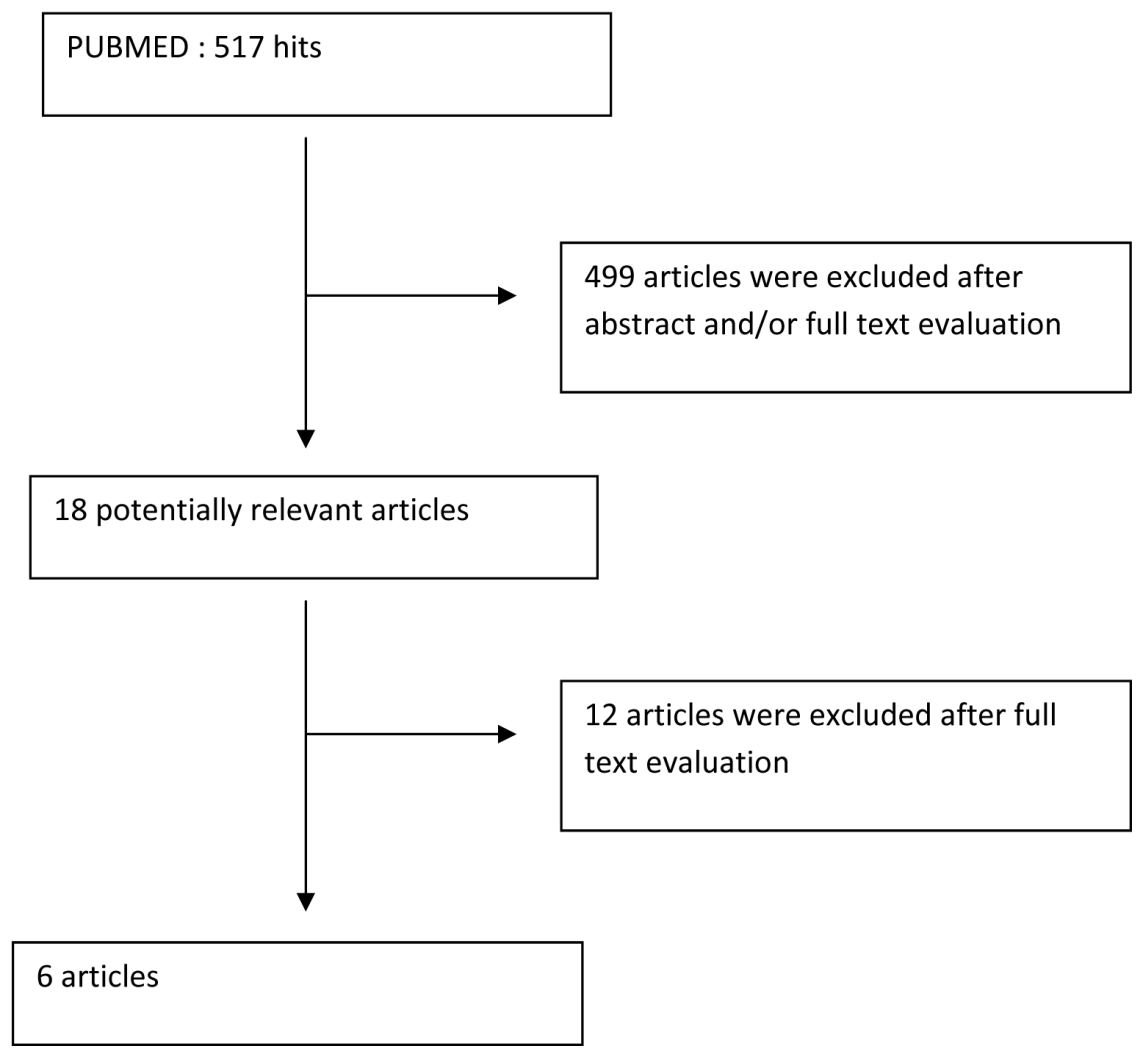
Search strategy, study selection, and study inclusion.

The initial strategy resulted in 517 articles, of which 18 were related to death following vaccination based on the abstracts and/or full texts. After reading the full texts, 6 papers pertaining to fatal anaphylaxis following vaccination were found relevant (Figure 1).

## RESULTS

We collected data on autopsy, histology, toxicology and biochemistry, when available in the selected articles (Table 1)

**Table 1:** Fatal presumptive anaphylactic cases following vaccine administration available in the literature

Authors, year of publication (ref. No.)	Age, gender	Administered vaccine Post-vaccination interval	Autopsy	Histology Immunohistochemistry	Biochemistry	Toxicology
Ziskind and Schattenberg, 1938 (10)	39 year-old female	Typhoid vaccine Second injection First symptoms 30 min post vaccination Death occurred shortly post vaccination	Petechial hemorrhages of the pleural surfaces Generalized congestion	Generalized congestion Compressed pulmonary alveoli with congested capillaries along with dilated alveoli and ruptured alveolar walls Eosinophils in the pulmonary vessels. Occasional eosinophils in the hepatic vessels	Not performed	Not performed
Walker, 1948 (11)	20 year-old male	Typhus vaccine Found dead 26 minutes after receiving typhus vaccine Allergic to eggs Yellow fever vaccine administered previously	No visible local reaction at vaccination location Generalized congestion Pulmonary edema	Generalized congestion Pulmonary edema	Not performed	Not performed
Werne and Garrow, 1946 (12)	10-month-old male identical twins	Second injection of diphtheria toxoid and pertussis Death within hours post vaccination	Petechial hemorrhages (several organs, including thymus) Pulmonary edema Generalized congestion	Numerous eosinophils in the lymphatic tissues, including the thymus Slight eosinophilic infiltration in bronchial and pulmonary artery wall	Not performed	Not performed
Curphey, 1947 (13)	3˝-year-old female	Influenza type A and B First symptoms 4 h post vaccination Death 7 h ˝ post vaccination	Generalized congestion Petechial hemorrhages (several organs, including thymus)	Occasional eosinophils in the lungs No eosinophils in the spleen Moderate eosinophil infiltration in lymph node	Not performed	Not reported
Pounder, 1983 (14)	33-year-old male	Vaccination against typhoid and cholera First symptoms 1 h post vaccination Death 8 h post vaccination	No visible local reaction at vaccination location No laryngeal edema Right atrial and ventricular dilatation	Occasional eosinophils sparsely scattered in the liver Cardiac conduction system unremarkable	Total IgE 720 IU/mL	Not reported
D’Errico et al, 2008 (9)	3-month-old female	Hexavalent vaccine Death 24 h post vaccination	Cardiac conduction system removed	Immunohistochemistry using anti-tryptase antibodies. Pulmonary mast cells and degranulating mast cell with tryptase-positive material outside identified. Cardiac conduction system unremarkable	Mast cell tryptase 43.3 μg/L	Negative

Mast cell tryptase was assessed exclusively in one case and revealed a concentration over the clinical reference values in the living. Total IgE concentration was measured in only one case, though without specific IgE and mast cell tryptase. Laryngeal edema was not detected in any of these cases whereas eosinophil or mast cell infiltration was observed in lymphoid organs including the spleen, liver and lungs. In one case, immunohistochemical investigations using anti-tryptase antibodies allowed pulmonary mast cells and degranulating mast cells with tryptase-positive material outside to be identified.

## DISCUSSION

An adverse event following vaccine administration is defined as any untoward medical occurrence following its administration and does not necessarily have a causal relationship with its use. Deaths following vaccination, especially when occurring in the first 48 h following vaccine administration, are of particular concern because they may raise community and health provider questions regarding the safety of the administered vaccine as well as the immunization program in general (15,16).

Four categories of suspected adverse events following vaccination have been reported: those induced by the injection process, those relating to a direct action of a vaccine component, those reflecting an immune-mediated process and those due to other mechanisms (17).

Adverse reactions reflecting an immune-mediated process include localized inflammatory responses, systemic inflammatory reactions, allergic (anaphylactic) reactions and other immune-related reactions. Vaccines are a mixture of compounds. Hence, allergic sensitization may occur to any component. Moreover, allergic reactions may follow administration of the first vaccine dose (7,15-18).

Vaccines include active immunizing antigens (complete microorganisms or their fragments, such as capsule polysaccharides), conjugating agents, preservatives, stabilizers, antimicrobial agents, adjuvant and culture media used in the preparation of the vaccine as well as the excipient used in the manufacturing process and inadvertent contaminants introduced during vaccine handling. Almost all vaccine components can be considered potential, allergic reaction triggers. These may include the natural rubber latex that can be contained in the syringe plunger as well as in the tips on prefilled syringes and vial stoppers. However, culture-derived proteins from eggs, gelatin (primarily used in viral vaccines to stabilize virus viability) and yeast are of particular importance. Other sources of allergic reactions are antibiotics and active immunizing antigens (7,9,15,18-23).

The mechanism involved in vaccine-associated allergies is generally considered to be a classical type I immediate hypersensitivity reaction involving an IgE-mediated response against a particular vaccine component. IgE-mediated reactions typically occur within minutes to an hour of exposure to relevant allergens and almost always within 4 hours of causative trigger contact. Type IV hypersensitivity delayed reactions have also been reported, though generally considered harmless. Type IV hypersensitivity reactions start 48 hours after vaccination and peak between 48 and 96 hours. They are typically observed with vaccines containing thimerosal (a mercury-containing preservative used in multidose vials that act as a preservative by inhibiting bacterial contamination), aluminum and antimicrobial agents in sensitized children and adults. Type IV reactions are becoming less frequent as mercury is being removed from modern vaccines. Type III hypersensitivity reactions have been described more rarely. These are attributed to the formation of circulating immune complexes between IgG antibodies and vaccine antigens and may induce a serum sickness-like disease or an exanthematous rash over 6-12 hours. Lastly, some delayed reactions may not be immunologically mediated. For instance, persistent hard nodules at the injection site have been attributed to local inflammatory reactions caused by irritant adjuvants such as aluminum and do not necessarily reflect immunologic hypersensitivity to vaccine constituents, though allergy to aluminum salts has also been proposed as an underlying mechanism (7,17,18,24,25).

Mast cells and basophils are the primary effector cells of classic type I immediate hypersensitivity reactions in humans. On the other hand, clinical and experimental experience has indicated that eosinophils are prominently engaged in allergic conditions and are implicated in the pathogenesis of anaphylaxis. The accumulation of eosinophils and mast cells in the spleen appears to be a hallmark of anaphylaxis, though it is still unclear whether splenic eosinophil infiltration is the consequence of recruitment through locally released chemotactic factors or whether it reflects general eosinophil increase in blood and tissue due to an allergic disposition. While the role of mast cells in human anaphylaxis is rather definite, issues such as the identification of the chemotactic factors that attract eosinophils in the spleen and the major signaling mechanism(s) for eosinophil activation remain poorly understood. Activated mast cells release numerous effector molecules, some of which could act as eosinophil chemotactic factors and consequently be responsible for eosinophil accumulation in the spleen (26-30).

The association between sudden infant death syndrome (SIDS), sudden unexpected death in infancy and vaccination has been discussed frequently. Because of the close temporal association between the first immunizations and the main peak of the SIDS incidence, it has been speculated that vaccinations may cause SIDS or sudden unexpected deaths in infancy. Nevertheless, several case-control studies have suggested that the apparent association between SIDS/sudden unexpected deaths in infancy and vaccinations occurs no more frequently than simply by chance or that immunizations may even be protective against SIDS. These results notwithstanding, the issue arises anew from time to time (16,25,31,32).

In 2003 a German study gave rise to suspicion of a possible association between hexavalent vaccine administration and sudden unexpected death in infancy. The signal was based on the observation of three deaths occurring between November 2000 and June 2003 in toddlers in their second year of life within 48 hours following administration of the fourth vaccine dose. In what became a heatedly discussed and criticized letter, another German team described six cases of sudden infant death shortly after hexavalent vaccination. In March 2011, Japan’s Health Ministry ordered doctors to stop immunizing infants with hexavalent vaccines subsequent to the sudden death of five babies within a short period of time after administration (33-39).

It has been emphasized that the definition itself of sudden infant death or sudden unexpected death in infancy following (hexavalent) vaccination is rather questionable. First, exhaustive postmortem examinations in presumptive vaccination-related death cases are not systematically performed. In addition, if examinations are performed, they may not include in-depth studies of the autonomic nervous system, vital centers of the brainstem or cardiac conduction system on serial sections. Morphological abnormalities in the brainstem and cardiac conduction system (such as arcuate nucleus hypoplasia in the brainstem as well as persistent fetal dispersion and resorptive degeneration in the cardiac conduction system) have been proposed in infants dying suddenly and unexpectedly as possible morphological substrates for sudden reflexogenic deaths, thus highlighting the importance of extensive postmortem investigations in all deaths occurring in infancy and perinatal age shortly after immunization (25,34,40).

As indicated above, vaccines are a mixture of compounds and almost all vaccine components can be considered as potential triggers to an allergic reaction. Anaphylaxis following vaccination is considered very rare with a documented risk in immunization information provided to the public of 1-2 cases per million vaccine dose. However, this becomes less clear when one considers the published literature on reported rates of anaphylaxis following vaccination. Indeed, this varies widely from 0.65 to 120 per million vaccines. The significant variation in the reported rate of anaphylaxis may be accounted for by a myriad of factors. Most importantly of these is case ascertainment and denominator data accuracy (ie, the number of vaccine doses administered). Case ascertainment will vary according to surveillance method (spontaneous reporting or active surveillance) and used case definition. Factors inherent to the target population being vaccinated as well as those inherent to the vaccine composition may also likely contribute to the variable rates of anaphylaxis (8,9,15,22-24,41).

The onus anaphylaxis detection as an adverse event following immunization falls into national, post-market surveillance systems, all of which rely on passive cases reporting. The reporting system of the Medicines and Healthcare Products Regulatory Agency in the UK received 130 reports of anaphylaxis associated with immunization in the six years from 1997 to 2003, suggesting a rate of 1 per million vaccine doses. Likewise, the US Vaccine Adverse Event Reporting System (VAERS) recorded 452 reports of reactions in over 1.9 billion doses of vaccines administrated countrywide over a 10-year period. This yields an estimated incidence of 0.2 cases for million vaccine doses. All post-marketing surveillance systems rely on passive case reporting and are thus prone to underreporting. These incidences are also of overall reaction rates and do not reflect incidences following individual vaccines (8,9,15,19,20).

Case descriptions of anaphylactic reactions to almost all vaccines exist. The incidence of anaphylactic reactions to the yellow fever vaccine reported to VAERS from 1991 to 1997 was about 1 per 131.000 doses. The reaction often occurred after the first vaccine dose, indicating that ingredients other than the immunizing vaccine antigen itself may have been the cause of the allergic reaction. Nagao et al. (22) demonstrated significantly increased influenza vaccine-specific IgE and vaccine-induced basophil activation in patients with influenza-vaccine induced anaphylaxis (IVA) who were included in the IVA spike in 2011-2012 in Japan. Judging by the number of case reports, the incidence of anaphylactic reactions to vaccines was higher 50 to 60 years ago than it is today. One reason for this may be that diagnostic criteria are more stringent nowadays than in the past. Another explanation can be that many of the reactions were caused by vaccine impurities. Indeed, improved purification processes have reduced the amount of allergenic substances in vaccines. Nevertheless, it can be said that severe systemic allergic reactions to vaccines were rare even 50 years ago (7-9,18,21-24,30,41,42).

Anaphylaxis-related deaths can present a series of unique challenges to forensic pathologists for several reasons. First, most forensic cases have incomplete, unreliable or absent medical records when bodies are admitted to the mortuary. Additionally, macroscopic and microscopic findings may be unspecific or absent. Furthermore, factors consistent with the hypothesis of fatal anaphylaxis include an immediately preceding challenge with an allergen known to cause reactions, clinical features consistent with or suggesting anaphylaxis, a previous history of reactions to similar or cross-reactive allergens, specific IgE antibodies to the allergen suspected of causing the reaction as well as measurable products of mast cell activation (26,43-45).

In the clinical setting, the diagnosis of anaphylaxis is based on consistent symptoms following exposure to potential triggering agents and may be further confirmed by increased levels of histamine and mast cell tryptase in plasma or serum. Unfortunately, practical consequences related to sampling times arise from the differing kinetics of histamine and mast cell tryptase appearance and elimination from blood. Indeed, samples for histamine determination should be obtained within 15 minutes of anaphylaxis onset, which might only be possible in a small proportion of cases and precluded in most of them. Conversely, samples for mast cell tryptase measurement can be obtained up to several hours after the reaction begins (26,44,46-49).

Based on the above, histamine determination is of no value for diagnostic purposes in the forensic setting whereas mast cell tryptase is. Indeed, mast cell tryptase levels can be assessed in postmortem serum even days after death and significantly increased postmortem serum tryptase levels have been reported repeatedly in cases of fatal anaphylaxis (26,44-47,50,51).

Although elevated postmortem serum tryptase concentrations may support the existence of mast cell activation, failure to document its elevation does not refute the diagnosis of anaphylaxis. Indeed, some cases of clinically diagnosed anaphylaxis to orally-ingested food allergens failed to show elevated tryptase levels, though abundant amounts of IgE against the ingested allergen could be detected. Moreover, beyond cases of fatal anaphylaxis, increased levels of postmortem serum tryptase have been observed in subjects with causes of death unrelated to anaphylaxis. This leads to the conclusion that postmortem, straightforward diagnoses of anaphylaxis cannot be exclusively based on mast cell tryptase determination alone (26,44,45,51-58).

Total and specific IgE antibodies appear to be relatively stable in postmortem serum samples and their measurements have proven useful for diagnostic purposes. However, total IgE determination in postmortem serum can only provide information pertaining to atopic disposition in individual cases. This means that increased levels do not prove that death was preceded by an IgE-mediated allergic reaction. In addition, similar to mast cell tryptase, increased total IgE levels have been observed frequently in situations unrelated to anaphylaxis, including diseases characterized by immune deficiencies or significant inflammatory components (26,44,45,59,60).

It has been proposed that combining results from mast cell tryptase determination in postmortem serum with a more specific assay for allergen sensitivity (such as allergen-specific postmortem serum IgE assays, if the identity of the allergen causing anaphylaxis is known or suspected) might support the hypothesis of IgE-mediated fatal anaphylaxis. Nevertheless, high levels of allergen-specific IgE exclusively indicate the degree of specific allergen sensitization in individual cases and do not prove death preceded by anaphylaxis. Hence, increased postmortem serum total and specific IgE levels cannot prove fatal allergic anaphylaxis (45,58,60-62).

Activated mast cell and eosinophil accumulation in the red pulp of the spleen has been demonstrated by several authors in anaphylaxis-related deaths using histochemical methods and specific immunohistochemical staining with monoclonal antibodies (44,54,63-66).

Today, a general consensus exists among researchers in recommending splenic tissue be systematically sampled for immunohistochemical investigations in all suspected anaphylaxis-related deaths. This is suggested in considering eosinophil and mast cell accumulation in splenic red pulp, along with increased postmortem serum tryptase levels, as the most reliable combination of microscopic findings and biochemical results to diagnose fatal anaphylaxis (44,65).

In addition, systematic histological examination of the myocardium and coronary arteries has been recommended by some authors. They suggest traditional and specific staining for mast cell identification in order to identify adventitial eosinophils and mast cells potentially involved in sudden death due to coronary artery spasm (67-72).

Very few cases of presumptive fatal anaphylaxis following immunization (including vaccine details, autopsy and histology findings as well as toxicology and biochemistry results when these analyses were performed) have been reported in the literature (9-14,73). The most exhaustively described case is the one by D’Errico et al. (9) in a 3-month-old baby who was administered hexavalent immunization in 2008. These authors performed not only biochemical analyses, which allowed an increased level of mast cell tryptase (43.3 μg/L) to be measured, but also immunohistochemical investigations using anti-tryptase antibodies, which allowed pulmonary mast cells and degranulating mast cells with tryptase-positive material outside to be identified. Moreover, these authors actually removed the cardiac conduction system during autopsy to carry out its histological examination. Examination of the system proved unremarkable.

The other reported cases, though interesting, lack either biochemical analysis or immunohistochemistry. Toxicology was only occasionally performed. Hence, the diagnosis of vaccination-related fatal anaphylaxis, though suggestive, remains (in our opinion) a mere hypothesis.

An analogous consideration may be applied to the case series reported by Zinka et al (35). These authors observed a slight eosinophil infiltration in various areas: in the liver in four out of six cases, in the lung in two out of six cases, in the spleen in just one case and at the cutaneous injection site in one case. Mast cell tryptase was measured in three out of six cases and levels were increased in two out of three. IgE levels were normal and specific IgE were assessed exclusively against tetanus toxoid and latex, both with negative results.

While the forensic literature on this topic is extremely scarce in comparison to other “allergic” or “anaphylactic” fatalities (which could lead to the conclusion that fatal anaphylaxis following vaccination is indeed an extremely rare occurrence, that these cases are only exceptionally investigated in the medico-legal setting or that they are rarely described in the literature as case reports), a very high numbers of papers exist in the clinical setting pertaining to vaccination-related deaths and other serious adverse vaccination-related events reported to various national, post-market surveillance systems. What is surprising “forensically” speaking, is that almost all these papers document a certain number of cases of vaccination-related deaths, though autopsy findings and data from death certificates are only available in a fraction of them (6,74-79).

If, on one hand, it is true that all post-marketing surveillance systems rely on passive case reporting and are prone to underreporting, on the other hand it is true that the number of more or less presumptive vaccination-related death cases (including presumptive fatal anaphylaxis cases following immunization) that had properly undergone in-depth forensic investigations is uncertain. In-depth refers to investigations characterized by histology, immunohistochemistry, biochemistry, toxicology and microbiology as well as microscopic examination of the cardiac conduction system and neuropathology with examination of the autonomic nervous system and vital centers of the brainstem in children. The question then arises, from a forensic point of view, about how many of these presumptive vaccination-related death cases or fatal anaphylaxis cases following immunization are actually caused by the administered vaccine.

Some years ago, the Brighton Collaboration Anaphylaxis Working Group developed case definition and guidelines for data collection, analysis and presentation for anaphylaxis as an adverse event following immunization. This very important paper stipulates that, because of uncertainties regarding the specificity of mast cell tryptase in the diagnosis of anaphylaxis and the absence of vaccine-specific data, mast cell tryptase measurement should be performed in suspected anaphylaxis cases, though this analysis should be considered exclusively as a minor diagnostic criterion. Where the presence of antigen specific serum-IgE does not necessarily predict clinical allergic manifestations, the absence of specific IgE does not rule out anaphylaxis. Hence, though specific IgE may have a useful role in causality assessment, it is not appropriate in case ascertainment. Lastly, the Group emphasizes that since anaphylaxis does not produce pathognomonic postmortem features, the proposed case definition of anaphylaxis should not take autopsy findings into account. On the basis of the conclusions of the Group, data from histology and immunohistochemistry are not considered in the case definition (80).

In conclusion, from a forensic point of view, all cases of sudden unexpected death in both adults and children require as many postmortem investigations as possible to be performed in order to rule out third party involvement and ascertain the cause of death as precisely as possible.

Regarding fatalities following drug administration, the role of forensic investigations is to determine whether the deaths are coincidental or causally related to the administered drug, irrespective of whether the drug is an antibiotic, contrast medium or vaccine. Death causally linked to drug administration but above all to obligatory vaccination may indeed give rise to social concerns, which must be avoided as far as possible, in the absence of reliable scientific evidence.

In suspected IgE-mediated fatal anaphylaxis, including fatal anaphylaxis potentially following vaccination, biochemical investigations should be systematically performed. However, it is worth emphasizing that increased postmortem serum levels of mast cell tryptase, total IgE and specific IgE do not prove (individually considered) that death was preceded by an IgE-mediate event. On the other hand, the absence of increased postmortem serum levels of mast cell tryptase does not allow the hypothesis of fatal anaphylaxis (following immunization) to be ruled out.

The accumulation of activated mast cells and eosinophils in the red pulp of the spleen has been repeatedly demonstrated in anaphylaxis-related fatalities. Consequently, splenic tissue should be routinely sampled for immunohistochemical investigations in all suspected anaphylaxis-related deaths and mast cell/eosinophil infiltrations should be systematically sought out in the spleen, myocardium and coronary artery wall.

Despite a certain number of uncertainties, eosinophil and mast cell accumulation in splenic red pulp, along with increased postmortem serum mast cell tryptase levels, can be considered the most reliable combination of microscopic findings and biochemical results in diagnosing fatal anaphylaxis in the forensic setting. Within the aforementioned limits, this same consideration should be applied to presumptive fatal anaphylaxis following immunization.

Microscopic evaluation of the cardiac conduction system and in-depth neuropathological examination including investigation of the autonomic nervous system and vital centers of the brainstem should always be performed in all cases of sudden unexpected deaths in infancy. This is especially important in situations potentially related to drug/vaccine administration in order to ascertain the role played by the administered medication.

On the other hand, all cases of sudden unexpected death in both adults and children require microbiological, toxicological and biochemical investigations. These are mandatory to exclude infection, intoxication and metabolic disturbances as causes of death.

To conclude, we are of the opinion that the hypothesis of fatal anaphylaxis following vaccination should be formulated exclusively when circumstantial data, medical records when available, and postmortem investigation results (mast cell tryptase, total IgE and specific IgE determination, immunohistochemical research of eosinophil and mast cell accumulation in splenic red pulp) converge in a consistent pattern. In addition, the reasonable exclusion of alternative causes of death is imperative in order to establish or rule out a cause-and-effect relationship between vaccine administration and any presumptive temporarily-related death.
